# Using machine learning to determine the correlation between physiological and environmental parameters and the induction of acute mountain sickness

**DOI:** 10.1186/s12859-022-04749-0

**Published:** 2022-05-31

**Authors:** Chih-Yuan Wei, Ping-Nan Chen, Shih-Sung Lin, Tsai-Wang Huang, Ling-Chun Sun, Chun-Wei Tseng, Ke-Feng Lin

**Affiliations:** 1 Graduate Institute of Life Sciences, National Defense Medical Center, No.161, Sec. 6, Minquan E. Rd., Neihu Dist., Taipei, 11490 Taiwan; 2 Department of Biomedical Engineering, National Defense Medical Center, No.161, Sec. 6, Minquan E. Rd., Neihu Dist., Taipei, 11490 Taiwan; 3 Department of Computer Science and Information Engineering, Chinese Culture University, No.55, Hwa-Kang Road, Yang-Ming-Shan, Taipei, 11114 Taiwan; 4 Division of Thoracic Surgery, Department of Surgery, Tri-Service General Hospital, National Defense Medical Center, No.325, Sec. 2, Chenggong Rd., Neihu Dist., Taipei, 11490 Taiwan; 5 School of Medicine, National Defense Medical Center, No.161, Sec. 6, Minquan E. Rd., Neihu Dist., Taipei, 11490 Taiwan; 6 Medical Informatics Office, Tri‑Service General Hospital, National Defense Medical Center, No.325, Sec. 2, Chenggong Rd., Neihu Dist., Taipei, 11490 Taiwan; 7 School of Public Health, National Defense Medical Center, No.161, Sec. 6, Minquan E. Rd., Neihu Dist., Taipei, 11490 Taiwan

**Keywords:** Acute mountain sickness, Physiological information, Lake Louise acute mountain sickness score, Blood oxygen saturation, Heart rate variability, Multivariate analysis

## Abstract

**Background:**

Recent studies on acute mountain sickness (AMS) have used fixed-location and fixed-time measurements of environmental and physiological variable to determine the influence of AMS-associated factors in the human body. This study aims to measure, in real time, environmental conditions and physiological variables of participants in high-altitude regions to develop an AMS risk evaluation model to forecast prospective development of AMS so its onset can be prevented.

**Results:**

Thirty-two participants were recruited, namely 25 men and 7 women, and they hiked from Cuifeng Mountain Forest Park parking lot (altitude: 2300 m) to Wuling (altitude: 3275 m). Regression and classification machine learning analyses were performed on physiological and environmental data, and Lake Louise Acute Mountain Sickness Scores (LLS) to establish an algorithm for AMS risk analysis. The individual R^2^ coefficients of determination between the LLS and the measured altitude, ambient temperature, atmospheric pressure, relative humidity, climbing speed, heart rate, blood oxygen saturation (SpO_2_), heart rate variability (HRV), were 0.1, 0.23, 0, 0.24, 0, 0.24, 0.27, and 0.35 respectively; incorporating all aforementioned variables, the R^2^ coefficient is 0.62. The bagged trees classifier achieved favorable classification results, yielding a model sensitivity, specificity, accuracy, and area under receiver operating characteristic curve of 0.999, 0.994, 0.998, and 1, respectively.

**Conclusion:**

The experiment results indicate the use of machine learning multivariate analysis have higher AMS prediction accuracies than analyses utilizing single varieties. The developed AMS evaluation model can serve as a reference for the future development of wearable devices capable of providing timely warnings of AMS risks to hikers.

## Background

Acute mountain sickness (AMS) is a maladaptation syndrome that occurs at high altitudes [1, 2]. The occurrence rate of AMS in Taiwan is 36% [3], and patient age and AMS risk are not correlated [4]. An altitude of 2500 m is considered the threshold for AMS [5]. Mountain sickness is mainly caused by the low oxygen concentration in mountain environments. Low oxygen concentrations increase sympathetic nervous system activity and subsequently cerebral blood volume, resulting in hypoxia [6, 7]. Every year, millions of mountaineers engage in activities at high-altitude locations, with some overlooking and or dismissing the risk of AMS. Studies have revealed that AMS may cause life threatening conditions, including but not limited to high-altitude pulmonary and cerebral edema [1].

As of this writing, the mechanism and diagnosis of AMS remain uncertain. The 2018 Lake Louise Acute Mountain Sickness (LLS) is currently used to assess the severity of AMS. The score is based on headache, gastrointestinal symptoms, fatigue and/or weakness, and dizziness or lightheadedness. The maximum total score is 12 points, with 3–5, 6–9, and 10–12 points indicating mild, moderate, and severe levels of AMS, respectively [8]. However, because these scores are susceptible to subjective bias, an objective scoring system is necessary for diagnosing AMS symptoms. Multiple studies have indicated that in high-altitude environments, the human body stimulates the sympathetic and parasympathetic nervous systems to moderate the heart rate to adapt to the low-pressure and low-oxygen environment. This process of changing heart rate is observable in electrocardiograms (ECGs) [9–12]. Therefore, heart rate variability (HRV) is a critical factor in AMS diagnosis [13–17]. Relevant studies have proposed that AMS may be caused by multiple factors, including environmental conditions (e.g., altitude, ambient temperature, and atmospheric pressure) and biological factors (e.g., heart rate, blood oxygen saturation (SpO_2_), and HRV) [2, 6, 18, 19]. Recent studies have used wearable devices to take physiological measurements; these studies measured users’ heart rate, SpO_2_, and HRV to diagnose mountain sickness [20, 21].

The majority of studies on AMS perform linear analyses to build AMS prediction models with environmental and biological data (e.g., SpO_2_ and HRV) that are collected daily. [13, 22, 23] As compared to linear analyses, multivariate analyses have higher model sensitivity, specificity, and accuracy; however, require increased quantities of data [24]. Therefore, to facilitate optimal training outcomes, multivariate analyses are performed on collected data in this study; and to ensure data is sufficient, environmental and physiological data are continuously and timely measured.

To summarize, this study employed machine learning algorithm applications to determine the correlation between AMS and environmental and physiological factors. The proposed algorithm can be used to predict the occurrence of AMS in real time. Furthermore, the algorithms can be applied to evaluate AMS risk among hikers, prevent the occurrence of hiking incidents, and ensure the safety of mountain activities.

## Results

### Coefficient of determination (R^2^) analysis

We used linear regression analysis of the LLSs and the recorded altitude, ambient temperature, atmospheric pressure, relative humidity, climbing speed, heart rate, blood oxygen saturation (SpO_2_), and heart rate variability (HRV) was performed using MATLAB R2020a to obtain the coefficient of determination (R^2^) of each variable and all variables. The coefficients of determination for altitude, ambient temperature, atmospheric pressure, relative humidity, climbing speed, heart rate, SpO_2_, HRV, and all variables were 0.1, 0.23, 1.2 × 10^–6^(≒0), 0.24, 1.5 × 10^–5^(≒0), 0.24, 0.27, 0.35, and 0.62, respectively. Single variable analysis revealed that HRV had the highest coefficient of determination, namely 0.35. The coefficient of determination for all eight variables was 0.62. The correlation analysis results are presented in Table 1. A comparison between the results of univariate and multivariate coefficient of determination analyses revealed that multivariate analysis yielded more satisfactory results. Therefore, this study employed the multivariate analysis results for subsequent binary classification analysis.

**Table 1:** R-squared results for the linear regression model

Variable	Linear Regression R-Squared	*p*
Altitude	0.1	< 0.001
Ambient temperature	0.23	< 0.001
Atmospheric pressure	1.2 × 10^–6^	< 0.001
Relative humidity	0.24	< 0.001
Rise rate	1.5 × 10^–5^	< 0.001
Heart rate	0.24	< 0.001
SpO_2_	0.27	< 0.001
HRV	0.35	< 0.001
Above all variables	0.62	< 0.001

SpO_2_: blood oxygen saturation. HRV: heart rate variability

### Binary classifier

**Fig. 1:**
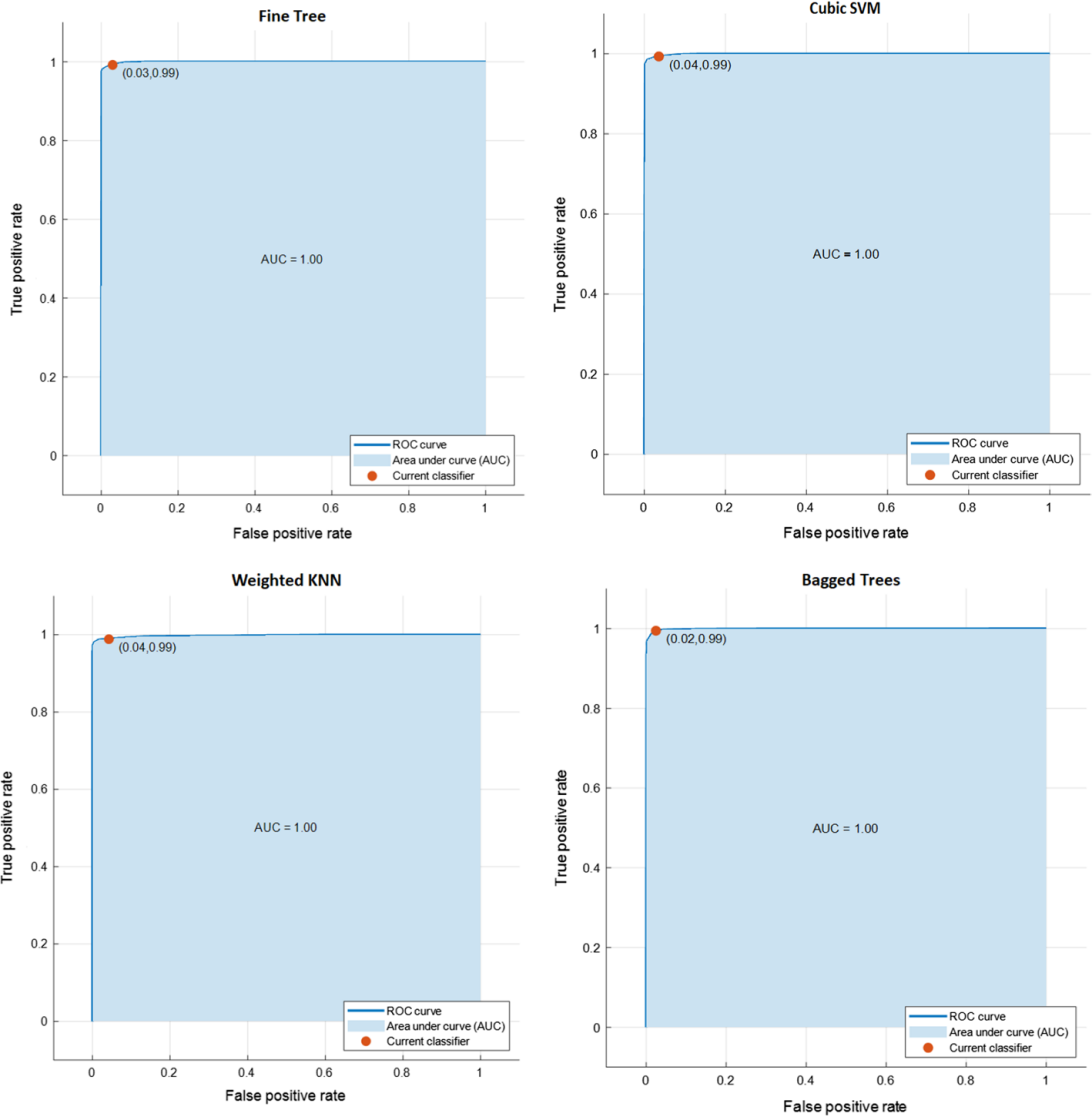
Area under the ROC curve for binary classifiers (Fine Tree, Cubic SVM, Weighted KNN, and Bagged Trees)

Based on the suggestion of The Lake Louise AMS Score Consensus Committee in the 2018, we considered an LLS of 3–5 to indicate mild AMS [8]. Therefore, we set an LLS < 3 as 0 and an LLS ≥ 3 as 1. The 25 machine learning algorithms employed the eight variables as the predictors to establish a machine learning model to diagnose mild AMS. The most favorable classification results were obtained by using bagged trees classifiers, achieving a sensitivity, specificity, accuracy, and area under the receiver operating characteristic curve (AUC) of 0.999, 0.994, 0.998, and 0.9999(≒1), respectively. See Table 2 for the classification results of each algorithm. Figure 1 shows the 4 algorithms: Fine Tree, Cubic SVM, Weighted KNN, Bagged Trees, which yielded the highest AUCs’ respective receiver operating characteristic (ROC) curves.

**Table 2:** Binary mild AMS classification results

Classifier type	Sensitivity	Specificity	Accuracy	AUC
*Decision Trees*				
Fine Tree	0.998	0.978	0.996	0.9999
Medium Tree	0.993	0.952	0.988	0.99
Coarse Tree	0.975	0.862	0.963	0.90
*Discriminant Analysis*				
Linear Discriminant	0.977	0.730	0.946	0.98
Quadratic Discriminant	0.997	0.707	0.952	0.99
*Logistic Regression Classifiers*				
Logistic Regression	0.978	0.858	0.965	0.99
*Naive Bayes Classifiers*				
Gaussian Naive Bayes	0.983	0.498	0.886	0.96
Kernel Naive Bayes	0.990	0.766	0.960	0.99
*Support Vector Machines*				
Linear SVM	0.981	0.858	0.967	0.99
Quadratic SVM	0.995	0.939	0.989	0.9999
Cubic SVM	0.997	0.967	0.994	0.9999
Fine Gaussian SVM	0.995	0.975	0.992	0.9999
Medium Gaussian SVM	0.995	0.914	0.985	0.9999
Coarse Gaussian SVM	0.974	0.895	0.966	0.98
*Nearest Neighbor Classifiers*				
Fine KNN	0.997	0.972	0.994	0.99
Medium KNN	0.996	0.957	0.991	0.9999
Coarse KNN	0.977	0.866	0.965	0.99
Cosine KNN	0.996	0.940	0.990	0.9999
Cubic KNN	0.995	0.949	0.990	0.9999
Weighted KNN	0.997	0.970	0.994	0.9999
*Ensemble Classifiers*				
Boosted Trees	0.998	0.984	0.997	0.9999
Bagged Trees	0.999	0.994	0.998	0.9999
Subspace Discriminant	0.970	0.795	0.951	0.97
Subspace KNN	0.997	0.959	0.993	0.9999
RUSBoosted Tree	0.999	0.929	0.991	0.9999

AUC: area under the receiver operating characteristic curve

The Bagged Trees yielded the highest sensitivity, specificity, accuracy, and AUC; and was bolded for that reason

## Discussion

In the results show that using MATLAB R2020a line regression analysis and machine learning model to diagnose mild AMS. We use measured environmental and physiological factors to Coefficient of determination (R^2^) for LLS. And the binary classification method using bagged trees classifiers to obtain a high degree of accuracy modeling, a variety of factors in the machine learning to build models, can be conducive to the development of the AMS risk assessment model.

The results revealed that the participants’ SpO_2_ measured at the start of the path was lower than 94, relatively lower than their mean SpO_2_ measured in their usual state at their home (SpO_2_: 97). In the experiment, the mean SpO_2_ was approximately 83.5 (in Table 2), indicating the occurrence of anoxia [25]. A comparison between with previous studies [19, 26] demonstrates that with an increase in altitude, the atmospheric pressure decreased, thereby causing a decrease in oxygen tension and resulting in the participants experiencing anoxia [19]. Therefore, SpO_2_ is a key indicator for diagnosing AMS.

Autonomic nervous system activity was monitored through HRV measurements; this method only requires observing R–R interval changes in the electrocardiogram (ECG). HRV are conducive to evaluating autonomic nervous system activity [9, 13, 14, 20] and produce fluctuations when the human body cannot adapt to low-pressure, low-oxygen environments; thereby indicating a decrease in the autonomic nervous system’s responsiveness [27].

In the measured results, the participants’ HRV significantly decreased in the mountain regions, thereby suggesting a correlation between the HRV-measured decrease in autonomic nervous system activity and anoxia severity, as indicated by SpO_2_. In high-altitude environments with low pressure and low oxygen, the autonomic nervous system response of patients with AMS decreases. Decrease in HRV demonstrates to more suggestive of AMS symptoms than decrease in oxygen levels. During the on-site experiment, some participants experienced an increased heart rate and expressed discomfort.

Relevant studies on the factors that influence AMS have revealed a low correlation between heart rate and LLSs. During the hiking process, the participants’ physiological indicators changed with hiking duration and altitude changes; in particular, participants experienced an increase in heart rate relative to their usual state.

The on-site experiment results indicate that in the hiking process, the variations of environmental factors resulted in changes in the participants’ physiological factors. Additionally, a positive correlation was revealed between heart rate and LLSs, whereas SpO_2_ and HRV exhibited a negative correlation with LLSs. This result is consistent with that of most studies on mountain sickness [9, 13, 20].

However, the coefficient of determination between atmospheric pressure and climbing speed with LLSs in this study was 0. Some studies have shown that the above two factors are related to AMS [9, 21]. This may be because this study only recorded data of participants engaging in hiking activities, and the elevation gain was slow; these two factors may have resulted in atmospheric pressure having a lower overall influence.

From the results, the 25 machine learning algorithms were employed to analyze the collected environmental and physiological factors, and demonstrated to yield sensitivity, specificity, and accuracy better than those of previous studies [13, 28], thus has comparatively higher developmental value.

## Limitations

The experiment location was at an altitude over 2500 m, thereby meeting the threshold for acute mountain sickness (AMS). However, because no participant exceeded six hours of hiking, the recorded data are considered short-term measurements. Additionally, the data may have been influenced by underlying factors, including the amount of exercise the participant had performed in the previous day and their rest and sleep duration. Therefore, this study had inadequate data when compared with studies that collected data for three consecutive days [13, 26, 28, 29]. These factors may have contributed to the differences in the measured physiological values [19, 23, 26, 30]. Furthermore, the unfavorable coefficient of determination of atmospheric pressure in linear regression may have been caused by the short experiment duration, which prevented the collection of a comparatively high number data points.

After participants put on the wearable devices and hiked from the Cuifeng Mountain Forest Park parking lot to Wuling, the ECG pads may have loosened due to participant activity, resulting in data inaccuracies. During data processing, we eliminated data collected during incidences when recording equipment was loosened to increase data accuracy. However, this also reduced the number of data collected and may have resulted in error values. In future studies, we plan to use more secure physiological monitoring equipment to prevent the occurrence of errors.

Participants were predominantly male and were relatively old in age. In the future, we will recruit more woman participants ranging between 18 and 40 years old and increase the data collection instances along the planned hiking path to increase the AMS diagnosis accuracy.

The highest LLS recorded in this study was 7, thereby indicating moderate AMS (LLS range: 6–9 points). During the measurement, the participant expressed immense discomfort and an inability to continue hiking. In accordance with institutional review board regulations, participants had the right to stop hiking. Therefore, this study did not collect data on participants experiencing severe AMS (LSS range: 10–12). This study focused on diagnosing whether AMS occurred. In the future, we will more thoroughly research all levels of AMS severity.

## Conclusions

When traveling to mountain regions, the low-pressure and low-oxygen environment may result in the weakening of the human heart self-regulating function. This increases the possibility of AMS or other high-altitude conditions. The literature on AMS suggests that among hikers exposed to simulated atmospheric pressure or low-pressure and low-oxygen conditions, those with lower SpO_2_ and HRV are more prone to experiencing AMS [31]. The analysis results of using environmental and physiological factors measured during on-site testing at over 2500 m altitude to predict AMS occurrence in participants revealed room for improvement.

We employed machine learning algorithms and various environmental and physiological factors for AMS diagnosis. Our model achieved a sensitivity and AUC of 0.99, indicating that the use of multivariate factors to train multivariate analysis-based machine learning models exhibit high developmental value in LLS evaluation. This machine learning algorithm can be coupled with equipment capable of the timely detection of multiple factor data to increase the accuracy for AMS predictions. Additionally, this application may prevent hikers from overlooking AMS symptoms and remind them to take preventive measures (e.g., taking medication or decreasing their altitude), thereby preventing the risk of severe AMS.

This study is the first to employ timely recorded environmental and physiological factors and multivariate analysis for AMS diagnosis. In terms of using LLS to diagnose AMS symptom severity, correlation analysis results revealed that the diagnosis effect of a single variable and LLS was unsatisfactory. However, the use of multiple variables and LLS exhibited satisfactory diagnosis effectiveness. The degree of correlation of SpO_2_ and HRV with AMS observed in this study was consistent with that of previous studies [32]. Additionally, correlation analysis results revealed that using more environmental and physiological factors with LLS may yield more satisfactory results. This finding is conducive to the establishment of AMS prediction and diagnosis models.

## Methods

### The pathogenesis and measurement methods of acute mountain sickness.

**Fig. 2:**
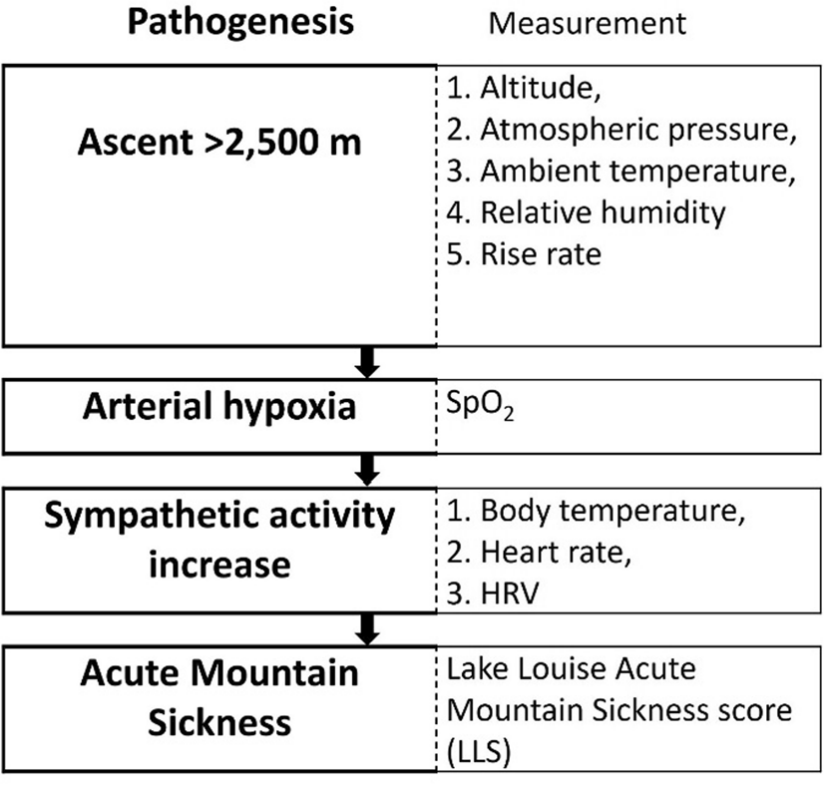
The flowchart for pathogenesis and measurement methods of Acute Mountain Sickness. This figure simply illustrates the pathogenesis of acute mountain sickness. In mountainous areas over 2500 m above sea level, the human body responds to measurable physiological factors in order to adapt to the alpine hypoxia. The thick bordered boxes show acute mountain sickness pathogenesis, the thin bordered boxes are the respective method of measurement

To research the physiological mechanisms of acute mountain sickness (AMS) [1, 2, 6], this study measured the environmental conditions (e.g., altitude, ambient temperature, atmospheric pressure, relative humidity, and climbing speed) and physiological variables (e.g., heart rate, blood oxygen saturation (SpO_2_), and heart rate variability (HRV) in real-time. The Lake Louise Acute Mountain Sickness score (LLS) was adopted to evaluate AMS severity. The flowchart (Fig. 2) for pathogenesis and measurement methods of Acute Mountain Sickness.

### Subjects

The experimental protocol was approved by the Tri-Service General Hospital Human Ethics Committee under registration number IRB: B202005136, and the informed consent of the experiment participants was obtained before this study. Adults between 25 and 55 years old capable of engaging in hiking activities and living below an altitude of 500 m were recruited as participants. Additionally, participants with diabetes, neuropathy, cardiovascular and pulmonary diseases, or other symptoms that could influence heart rate variability (HRV) were excluded. Before testing, participants were required to administered the 2017 Physical Activity Readiness Questionnaire (2017PAR-Q +) [33] to confirm if any of the aforementioned diseases were present and whether the participants used drugs that may influence HRV, including plant-based neuromuscular-blocking drugs and sedative drugs.

### Participant demographics

This study initially recruited 34 participants, however, only 32 fulfilled the inclusion criteria; two participants with self-reported cardiovascular disease and diabetes respectively were excluded. The final sample set were comprised of 25 men and 7 women. The mean participant age was 36.5 ± 8.1 years, and the mean body mass index (BMI) was 24.3 ± 2.36. The mean home altitude of the participants was 65.4 ± 90.4 m. No participant had consumed drugs (e.g., acetazolamide) 30 days before the experiment. The most common acute mountain sickness (AMS) symptoms were weakness and dizziness, which were observed in 13 (40.6%) of the participants. The baseline demographics of the participants are listed in Table 3.

**Table 3:** Baseline demographics

Demographic	Result
Age, y	36.5 ± 8.1
*Gender*	
Male	25 (78%)
Female	7 (22%)
Body weight, kg	67.4 ± 6.9
Body height, m	1.66 ± 0.07
BMI, kg/m^2^	24.3 ± 2.36
Home altitude, m	65.4 ± 90.4
*drug use within 30 day of ascent*	
Acetazolamide	0(0%)
Steroids	0(0%)
Asthma medication	0(0%)
Pain reliever	0(0%)
Smoker	2(6.3%)
Alcohol consumer	0(0%)
History of AMS	5(15.6%)
Knowledge of AMS	14(43.8%)

Values are presented as mean ± standard deviation. *BMI* body mass index

### Physiological and environmental variable sensors and measurement methods

This study used the MD-670P Plus portable pulse oximeter and electrocardiogram (ECG) monitor (Department of Health Medical Device Manufacturing No. 001657, COMDEK Industrial Corp., Taiwan) to measure heart rate, blood oxygen saturation (SpO_2_), and ECG. The monitor was connected to a Smart Link I application and a USB interface to transmit the data to a personal computer for data observation and analysis. Time domain analysis was performed on the collected ECG data, and the standard deviation of the R–R intervals was calculated to obtain the HRV data [15].

**Fig. 3:**
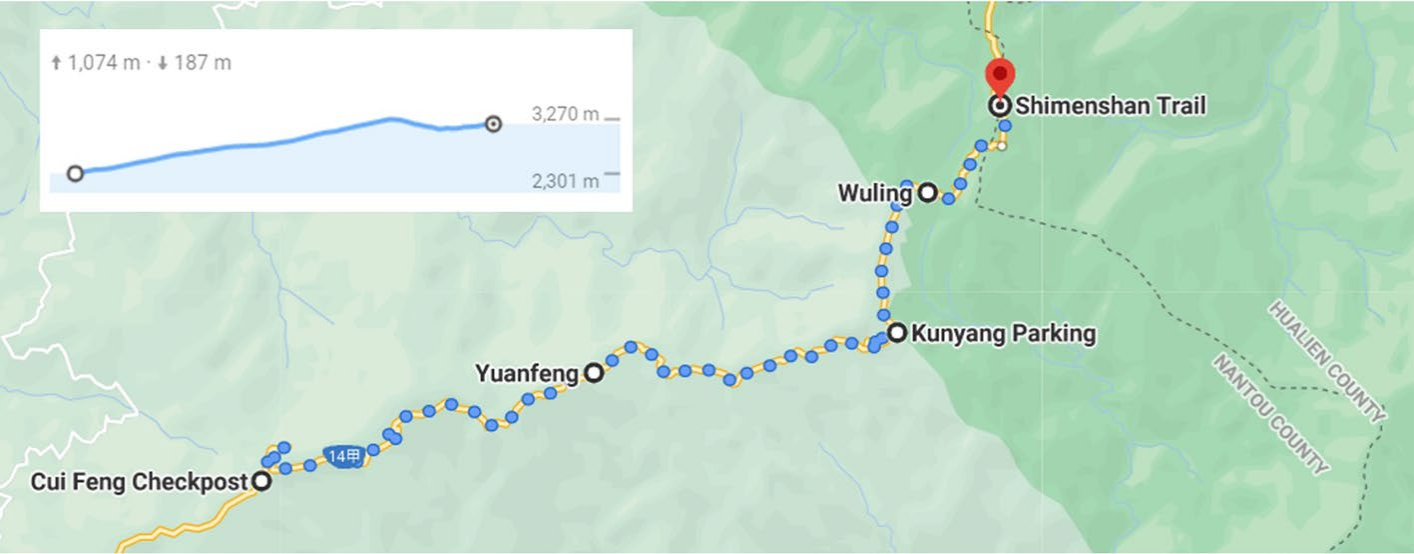
The map of the experimental route

Additionally, the TI CC2650 SensorTag (Texas Instruments, Texas, USA) was employed to measure the environmental factors of a participant’s position, including altitude, ambient temperature, atmospheric pressure, relative humidity, and climbing speed. Throughout the experiment, the participants were required to wear the pulse oximeter and ECG monitor and the SensorTag and hike along Provincial Highway 14. The participants embarked from the Cuifeng Mountain Forest Park parking lot in Nantou (2300 m altitude), passed the Yuanfeng Lookout (2756 m altitude), the Kunyuan parking lot (3085 m altitude), Wuling (3275 m altitude), and arrived at Shimen Mountain Class 3 Triangulation Point No. 6389 (3237 m altitude). The total hiking distance was 16.3 km, and the experiment duration was 8 h. The map of the experimental route is presented in Fig. 3 If the participants experienced swelling, itching, or other irritations where the wearable device was worn, they were required to immediately remove the device and were excluded from the experiment.

### Participants’ physiological variables

The participants’ physiological variables were collected from the heart rate, SpO_2_, and ECG records of the portable pulse oximeter and ECG monitor. During the hiking activity, participants who experienced discomfort were immediately required to complete the online digital LLS. Additionally, professionals were assigned to the Cuifeng Checkpost, Yuanfeng, Kunyang parking, Wuling, and Shimenshan Trail to verbally administer the LLS to passing participants, thereby preventing the participants’ subjective factors from influencing the acute mountain sickness assessment. The recording equipment automatically measured and recorded the data without requiring manual operation by the participants.

In the experimental process, data were not recorded during incidences when recording equipment (ECG pads and pulse oximeter) were loosened; a total of 8410 data points were collected and used for analysis. Table 4 presents the measured environment and participants’ physiological variables. The average heart rate, SpO_2_, and HRV values of participants in mountainous areas were 115.84, 83.5, and 39.17, respectively; and were slightly lower than in their usual state. The measured physiological variables of the participants engaging in hiking activities were roughly equal to those recorded in previous studies [14–17].

**Table 4:** Environment and participants’ physiological variables

	Altitude	Ambient temperature	Atmospheric pressure	Relative humidity	Rise rate	Heart rate	SpO_2_	HRV	LLS
Average	2868.79	24.26	698.11	0.64	3.08	115.84	83.5	39.17	0.64
Median	2852.89	24	693.36	0.67	3.96	117	84	38.9	0
Mode	3275	25	675.59	0.74	0	132	86	36	0
Std. Dev	297.59	1.73	18.9	0.12	3.4	19.32	4.92	10.76	1.3
Variance	88,562.6	3	357.16	0.01	11.6	373.14	24.18	115.71	1.7
Mini	2300	20	675.59	0.42	-10	77	70	13	0
Max	3275	29.4	742.2	0.78	10	160	94	80.5	7
Reliability (95%)	6.36	0.04	0.4	0.002	0.07	0.41	0.11	0.23	0.03

*Std. Dev* Standard Deviation

### Classification and evaluation

The MATLAB R2020a machine learning and deep learning tool (MathWorks, Natick, MA) [34] was employed for data analysis.

First, the linear regression model of regression learner applications was adopted to analyze the measured environmental and physiological factors. The coefficient of determination (R^2^) of each individual factor and all factors was calculated to determine factor correlations with the LLSs.

Subsequently, binary classification analysis was performed on the factors and the LLSs. The established mild AMS diagnosis model employed MATLAB R2020a’s classification machine learning application that used the eight collected physiological and environmental variables as predictors, and the classification output was set as whether a participant experienced mild AMS symptoms.

The classifier type selected in this study includes 25 machine learning algorithms such as: Decision Trees (Fine Tree, Medium Tree, Coarse Tree), Discriminant Analysis (Linear Discriminant, Quadratic Discriminant), Logistic Regression Classifiers (Logistic Regression), Naive Bayes Classifiers (Gaussian Naive Bayes, Kernel Naive Bayes), Support Vector Machines (Linear SVM, Quadratic SVM, Cubic SVM, Fine Gaussian SVM, Medium Gaussian SVM, Coarse Gaussian SVM)、Nearest Neighbor Classifiers (Fine KNN, Medium KNN, Coarse KNN, Cosine KNN, Cubic KNN, Weighted KNN), Ensemble Classifiers (Boosted Trees, Bagged Trees, Subspace Discriminant, Subspace KNN, RUSBoosted Tree).

Finally, the characteristics of the developed AMS prediction model were evaluated based on sensitivity, specificity, accuracy, and area under the receiver operating characteristic curve (AUC). To prevent training and or testing bias, the 8410 data points were randomly divided into 90: 10 training and testing sets respectively. The aforementioned 25 machine learning algorithms were used to build the model, and were validated with the tenfold cross validation to prevent reliance on sample characteristics; and also to stabilize the final model.

### Statistical analysis

After a classification algorithm has trained on data, we want to examine the performance of the algorithm on a specific test dataset. We assessed the predictive performance of our model using a range of common performance metrics: sensitivity, specificity, accuracy, and area under the curve (AUC), all of which range from 0 to 1 [35]. From Table 5, estimated sensitivity is the proportion of subjects with the condition of the true positives that are diseased. Estimated specificity is the proportion of subjects without the condition of true negatives that are diseased-free [36]. Accuracy is a commonly applied metric from the machine learning domain.

**Table 5:** Confusion matrix for binary classification

	Diseased	Diseased-free
Positive diagnostic test	True positives (TP)	False positives (FP)
Negative diagnostic test	False negatives (FN)	True negatives (TN)

Where TP = number of true positive events, FP = number of false positive events

TN = number of true negative events, FN = number of false negative events

The three conventional evaluation indicators (sensitivity, specificity and accuracy) were calculated as follows.

Sensitivity = TP/(TP + FN).

Specificity = TN/(FP + TN).

Accuracy = (TP + TN)/(TP + FN + FP + TN).

We can adjust different thresholds to get the true positive rate versus false positive rate (equivalently, sensitivity versus 1–specificity), by plotting a Receiver Operating Characteristic curve (ROC) to get the area under the curve (AUC) size, we can use the ROC curve to find the classifier that maximizes the classification accuracy, or to evaluate the performance of the classifier in high sensitivity and high specificity regions. We adopted tenfold cross-validation to obtain the mean values and standard deviations of these evaluation indicators to fairly compare their performance. In detail, the dataset is divided into ten approximately equal-sized sub-datasets, and the positive samples are divided equally in each sub-dataset.

## Data Availability

The data that support the findings of this study are available on request from the corresponding author. The data are not publicly available due to privacy or ethical restrictions.

## Abbreviations

AMS: Acute mountain sickness
LLS: Lake Louise acute mountain sickness scores
SpO_2_: Blood oxygen saturation
HRV: Heart rate variability
ECG: Electrocardiogram
2017PAR-Q +: The 2017 physical activity readiness questionnaire
BMI: Body mass index
SVM: Support vector machines
KNN: K-nearest neighbors
AUC: Area under the receiver operating characteristic curve
ROC: Respective receiver operating characteristic curve
TP: True positives
FP: False positives
FN: False negatives
TN: True negatives (TN)
